# Awareness of benign paroxysmal positional vertigo in central Israel

**DOI:** 10.1186/1471-2377-9-17

**Published:** 2009-04-22

**Authors:** Lea Pollak

**Affiliations:** 1 Department of Neurology, The Assaf Harofeh Medical Center, 730 00 Zerifin, Israel; 2 Sackler Faculty of Medicine, Tel Aviv University, Tel Aviv, Israel

## Abstract

**Background:**

Despite its frequent occurrence and effective treatment options, benign paroxysmal positional vertigo (BPPV) still remains under-estimated in the community.

**Methods:**

We reviewed referral letters and medical records of 120 patients who were treated for BPPV at our Dizziness Clinic during the years 2006–2008 and searched for factors that possibly contribute to missing this entity.

**Results:**

The referral diagnosis could be clustered into four groups: BPPV (25.6%), further unspecified vertigo (36.6%), dizziness (27.5%) and other (10%). BPPV was recognized more frequently by ENT doctors than by other specialists.

Patients referred with the correct diagnosis of BPPV were significantly younger and the duration of their symptoms shorter than in other referral groups. Patients in the distinct referral groups did not differ in the presence of autonomic symptoms or a history of another serious disease. A history typical of BPPV could be obtained in all but 11 patients, but position dependence was noted by the referring physician only in 55 patients, 31 of them correctly assigned as possible BPPV. Only in two patients was the Dix-Hallpike test performed. Thirty two patients were diagnosed with BPPV in the past, but this did not influence the recognition of the recurrence of this clinical entity. About 40% of patients had an audiogram and/or brainstem auditory evoked potentials. Electronystagmography was performed in 7.5% and brain imaging in 14% of patients before referral.

**Conclusion:**

Our results show that BPPV is still an under-recognized entity.

Education and the demand on specialists to learn how to treat BPPV, could improve the situation.

## Background

Benign paroxysmal positional vertigo (BPPV) is probably the most common diagnosis at vertigo clinics [1]. It is characterized by rotational vertigo induced by head position changes. Patients typically complain about attacks of vertigo when extending or turning the neck, getting up or lying down, or rolling over in bed. The attacks are often accompanied by a feeling of unsteadiness and loss of confidence during walking. The diagnosis is confirmed by the Dix-Hallpike positioning testing, or the roll test in cases of the horizontal canal variant BPPV. Particle repositioning maneuvers represent an effective treatment for BPPV, which leads to resolution of the positioning nystagmus in 70 to 100% of cases [2-5].

However, BPPV remains still under-diagnosed, among general practitioners and even among neurologists and ENT specialists [6-10].

We reviewed the referral diagnosis of BPPV patients at our clinic to search for factors that possibly contribute to missing this entity.

## Methods

We reviewed the referral letters and medical records of 120 patients who were treated for BPPV at our Dizziness Clinic during the years 2006–2008.

The clinic is situated in central Israel about 10 km from Tel Aviv. The patients are referred from the surrounding towns, villages and kibbutzim.

The diagnosis was based on a history of recurrent positional vertigo and the presence of a geotropic torsional nystagmus directed towards the undermost ear on Dix-Hallpike testing (posterior canal variant), or the presence of a geotropic/apogeotropic nystagmus on roll testing (horizontal canal variant of BPPV). One hundred and nine patients suffered from idiopathic and 11 from posttraumatic BPPV. In 100 patients, the posterior canal was involved and they were treated by the Epley maneuver. In 20 patients who had the horizontal canal type of BPPV, a barbecue treatment was applied.

Patients with bilateral BPPV or anterior canal BPPV were excluded.

The referral letters were reviewed in respect to the specialty of the provider and the putative diagnosis. The patients were characterized by age, sex and the presence of other serious diseases in their medical history such as oncological, severe internal or surgical diseases. The time period from onset of the present BPPV attack until treatment as well as the presence of vegetative symptoms such as severe nausea, vomiting, sweating, pallor, syncope or diarrhea, were registered. Patients were classed as "typical" in cases where a history of typical, positioning-induced, recurrent vertigo of short duration was obtained. Patients who complained mainly of unsteadiness, lightheadedness, headache or neck pain and where the positional component was less prominent were classed as "atypical". In these cases, the diagnosis was usually not suspected during history taking, but was revealed on testing.

Patients who were diagnosed and treated for BPPV at our clinic or elsewhere in the past were registered. Audiological studies and/or imaging studies performed prior to referral were noted.

Statistical methods included the chi-test for categorical variables and the Student's t-test for continuous variables. An error rate of less than 0.05 was considered significant for the p-value. The statistical analyses were performed using a commercially available software program (SPSS, version 10.0).

The study was approved by the local ethic committee of the hospital.

## Results

Sixty one percent of patients were referred by a family doctor, 7% by internists, 25% by ENT specialists, 3% by neurologists and 2% by neuro- or orthopedic surgeons.

**Figure 1:**
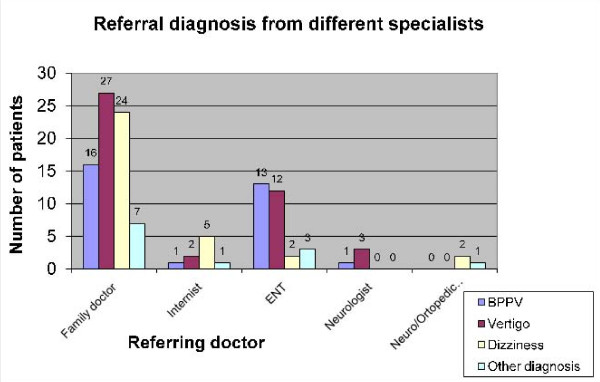
**Referral diagnosis from different specialists**.

The referral diagnosis could be clustered into four groups: BPPV (25.6%), further unspecified vertigo (36.6%), dizziness (27.5%) and other (10%). The latter included dysequilibrium (4 patients), headache (2), syncope (1), concentration problems (1), ear pain (1) and observation (3) (Table 1). BPPV was recognized more frequently by ENT doctors than by others. The referral diagnosis by different specialists can be seen in Figure 1.

**Table 1:** Data of BPPV patients in the distinct referral groups

	**Referral group (No. of patients)**						
	
**Patients' data**	**BPPV (31)**	**Unspecified (44)**	p*	**Dizziness (33)**	p*	**Other (12)**	p*
Age (years)	51.1 ± 15.3	59.6 ± 5.5	0.01	59.5 ± 16.3	0.01	54.4 ± 16.4	ns*

Sex (men)	10	8		9		4	

Duration (months)	1.3 ± 1	5.8 ± 9.8	0.02	3.8 ± 6.1	0.01	2.3 ± 3.3	ns

Recognition of position dependence**	100%	32%	0.000	24%	0.000	17%	0.000

Autonomic symptoms	32%	41%	ns	45%	ns	8%	ns

Typical history***	97%	86%	ns	91%	ns	83%	ns

Brain imaging	6%	14%	ns	12%	ns	42%	0.005

Audiogram	35%	32%	ns	52%	ns	50%	ns

BERA	32%	27%	ns	48%	ns	50%	ns

ENG	0%	7%	ns	12%	0.04	17%	0.01

BPPV in the past	26%	20%	ns	42%	ns	8%	ns

Other severe illness	39%	52%	ns	58%	ns	33%	ns

* p – The group vs BPPV, ns – non significant

** Recognition of position dependence of vertigo by the referring doctor

*** Typical history of BPPV as obtained at the Dizziness clinic

The mean age of patients referred under the heading of BPPV was significantly lower than the age of patients with another diagnosis on referral (Table 1). The duration of symptoms of the present attack of BPPV was found to be significantly shorter in patients with BPPV than in patients with other referral diagnoses (Table 1). Patients in the individual referral groups did not differ in the presence of autonomic symptoms or the existence of another serious disease.

Despite the fact that a history typical for BPPV could be obtained in all but 11 patients, position dependence was noted by the referring physician in only 55 patients, 31 of them correctly assigned as possible BPPV. Only two referring physicians performed the Dix-Hallpike test.

Thirty two patients were diagnosed with BPPV in the past, but this did not influence the recognition of the recurrence of this clinical entity.

Forty percent of patients had an audiogram and 36% had a brainstem auditory evoked potentials test (BERA) prior to the visit at the dizziness clinic. Electro-nystagmography (ENG) was performed in 9 patients (7.5%) suffering from dizziness and vertigo. None of the patients suspected to suffer from BPPV underwent an ENG.

Brain imaging (head CT or MRI) was performed in 17 patients (14%), more frequently in the dysequilibrium group than in other referral groups.

We identified 8 patients who were referred with the assumed diagnosis of BPPV and turned out to suffer from nonorganic vertigo (7 patients), or orthostatic hypotension (1 patient).

## Discussion

In our BPPV patient cohort, the correct diagnosis was suspected by the referring physician only in 25% of cases, most often by ENT doctors. The presence of autonomic symptoms or the history of another severe unrelated disease did not influence the referral diagnosis. It is surprising that earlier diagnosis of BPPV did not improve the recognition of the recurrence of this clinical entity. Forty five percent of the referring physicians noted a position-dependence or influence of vertigo during history taking. This led to the correct diagnosis in 56% of them. Only two physicians (both ENT) performed the diagnostic Dix-Hallpike maneuver and in one of them a particle repositioning maneuver was tried.

The mean age and symptom duration of patients who were referred under the heading of BPPV was significantly lower than the age and symptom duration of patients in other referral groups. All physicians who correctly suspected BPPV recognized the position-dependence of the symptoms. This might explain why in this group the referral time was shorter.

Elderly patients with BPPV often ignore the positional component of their symptoms and complain mainly about disequilibrium [10,11]. Moreover, elderly patients often have neck stiffness or other musculoskeletal problems which interfere with the performance of the Dix-Hallpike test. This might be the reason why BPPV was recognized more often in younger patients.

About 40% of BPPV patients had an audiogram and BERA before being seen at the dizziness clinic. These audiological tests are cheap, but redundant for establishing the diagnosis of BPPV. Moreover, ENG testing which is more expensive, less available and uncomfortable for the patient and can easily miss the diagnosis, was performed in 9 patients with BPPV. Remarkably, in all patients who had ENG, BPPV was not recognized. Brain imaging, which does not contribute to the diagnosis and may involve considerable side effects (i.e. radiation), was ordered in 14% of BPPV patients.

Our results clearly show that BPPV is still an under-recognized entity. In view of its frequency, the disability and emotional impact that it has on the patient, but mainly in view of the availability of simple and costless treatment options, our findings deserve attention.

We assume that the main reason for under-diagnosis of BPPV among general physicians (specialists in family or internal medicine) is the lack of familiarity with this entity. The benign course and spontaneous remissions could be other contributory factors for the relative unawareness of BPPV among community doctors [12,13]. Education in the form of lectures, courses or demonstration seminars, could improve the knowledge of the pathogenesis, tendency for recurrence and diagnostic tools for BPPV in this large group of first line physicians [14]. We think that treatment of BPPV by general practitioners would be out of the scope and possibilities of their work, also in view of the various forms of this clinical entity. Today there are a considerable number of vestibular physiotherapists who have learned how to treat BPPV in our country. We think, however, that the diagnosis should be made by a person with medical education, and not by the physiotherapist, as in patients with other medical conditions referred for physiotherapy. We see therefore the role of the general physician in the correct recognition of BPPV and their further referral to a vestibular physiotherapist. Patients with frequent recurrence of BPPV could be then instructed by the physiotherapist about self-treatment. Patients who do not provide the typical history and those in whom the general physician does not suspect the diagnosis of BPPV, would be referred to an ENT specialist or a neurologist.

The fact that ENT doctors recognized BPPV more often than others reflects their familiarity with this entity. It is possible that some ENT doctors treat their patients at the clinic or refer them to a trained physiotherapist. However, a large portion of ENT specialists (thirty in our study) did not cope with BPPV and preferred to refer the patients to a dizziness clinic. The number of patients referred by neurologists or neuron/orthopedic surgeons was too small to enable us to draw conclusions.

## Conclusion

It is our opinion that every ENT specialist, as well as neurologist, should be able to recognize and treat BPPV, at least the typical cases [15-18]. A referral to specialized dizziness clinics, audiological tests, ENG or brain imaging, should be kept for atypical or treatment resistant cases. To achieve this, theoretical knowledge is apparently not sufficient and the ENT or neurologist should receive training for BPPV treatment during their specialization as a part of the educational program. This should be regarded as being as important as the acquisition of operation skills among ENT residents or performing a lumbar puncture among specializing neurologists.

## Competing interests

The author declares that they have no competing interests.

## Pre-publication history

The pre-publication history for this paper can be accessed here:


